# Real-world effectiveness of ixazomib combined with lenalidomide and dexamethasone in relapsed/refractory multiple myeloma: the REMIX study

**DOI:** 10.1007/s00277-023-05278-3

**Published:** 2023-06-10

**Authors:** M. Macro, C. Hulin, L. Vincent, A. Charvet-Rumpler, L. Benboubker, C. Calmettes, A.-M. Stoppa, K. Laribi, L. Clement-Filliatre, H. Zerazhi, F. Honeyman, V. Richez, F. Maloisel, L. Karlin, J. Barrak, C. Chouaid, X. Leleu

**Affiliations:** 1 IHBN - CHU de Caen, Caen, France; 2 CHU Bordeaux - Hôpital Haut Leveque, Pessac, France; 3 CHU de Montpellier - Hôpital Saint-Eloi, Montpellier, France; 4 CHU de Besançon - Hôpital Jean Minjoz, Besançon, France; 5 CHRU de Tours - Hôpital Bretonneau, Tours, France; 6 CH de Périgueux, Périgueux, France; 7 Institut Paoli Calmettes, Marseille, France; 8 CH Le Mans, Le mans, France; 9 Clinique Louis Pasteur, Essey-lès-Nancy, France; 10 CH d’Avignon, Avignon, France; 11 CHU de Saint-Etienne, Saint-Etienne, France; 12 CHU de Nice - Hôpital de l’archet, Nice, France; 13 Clinique Sainte-Anne, Strasbourg, France; 14 Hospices Civils de Lyon, Pierre Bénite, France; 15 Takeda France, Paris, France; 16 CHI de Créteil, Créteil, France; 17 CHU de Poitiers, Poitiers, France

**Keywords:** Relapse and refractory multiple myeloma, Real world evidence, Ixazomib, Effectiveness, Safety, Elderly

## Abstract

Ixazomib (IXA) is an oral proteasome inhibitor (PI) used in combination with lenalidomide and dexamethasone (IXA-Rd) for patients with relapsed and/or refractory multiple myeloma (RRMM). The REMIX study is one of the largest prospective, real-world analysis of the effectiveness of IXA-Rd in the setting of RRMM. Conducted in France between August 2017 and October 2019, the REMIX study, a non-interventional prospective study, included 376 patients receiving IXA-Rd in second line or later and followed for at least 24 months. Primary endpoint was the median progression-free survival (mPFS). Median age was 71 years (Q1-Q3 65.0 – 77.5) with 18.4% of participants older than 80 years. IXA-Rd was initiated in L2, L3 and L4 + for 60.4%, 18.1% and 21.5%, respectively. mPFS was 19.1 months (95% CI [15.9, 21.5]) and overall response rate (ORR) was 73.1%. mPFS was 21.5, 21.9 and 5.8 months in patients receiving IXA-Rd as L2, L3, L4 + respectively. Among patients receiving IXA-Rd in L2 and L3, mPFS was similar for patients previously exposed to lenalidomide (19.5 months) than for those lenalidomide naive (not exposed, 22.6 months, *p* = 0.29). mPFS was 19.1 months in patients younger than 80 years and 17.4 months in those 80 years or older (*p* = 0.06) with similar ORR (72.4% and 76.8%) in both subgroups. Adverse events (AEs) were reported in 78.2% of patients including 40.7% of treatment-related AE. IXA discontinuation was due to toxicity in 21% of patients. To conclude, the results of the REMIX study are consistent with the results of Tourmaline-MM1 and confirm the benefit of IXA-Rd combination in real life. It shows the interest of IXA-Rd in an older and frailer population, with an acceptable effectiveness and tolerance.

**Supplementary information:**

The online version contains supplementary material available at 10.1007/s00277-023-05278-3.

## Background

Among the many treatments available for patients with relapsed/refractory multiple myeloma (RRMM), new, promising therapies have recently emerged [1, 2]. These new therapies offer patients further therapeutic options to respond to inevitable relapses during disease evolution [3].

Ixazomib (IXA) is the first orally-administered in its class. It has been approved in Europe and in the USA combined with lenalidomide and dexamethasone (Rd) for treating RRMM after first-line treatment, based on the results of TOURMALINE-MM1 phase 3 clinical trial [4]. TOURMALINE-MM1 was conducted in a population of patients having received in median 1 prior line of treatment (1–3) and demonstrated a significant longer median progression-free survival (PFS) with ixazomib-Rd (IXA-Rd) than with placebo-Rd (20.6 versus 14.7 months; hazard ratio [HR] 0.74, *P* = 0.01) and a significant increased overall response rate (ORR) with limited additional toxicity and a maintained level of quality of life [5].

As with many novel chemotherapeutic agents, the choice to prescribe IXA is based on finding a balance between efficacy, toxicity, and patient characteristics including age, frailty, or cytogenetic abnormalities. At early relapse, treatment choice is majorly orientated by refractoriness to lenalidomide and / or bortezomib. In an elderly population, assessment of frailty and comorbidities is also highly weighting on treatment choice [6–9]. In the setting of a frail population, the availability of a fully oral combination can be of great interest. Additionally, it has been estimated that a substantial proportion of typical RRMM patients (approximately 40%), are excluded from clinical trials, which makes translating clinical development results to real-life practice uncertain [9–11]. Thus, defining the most appropriate treatment sequences for each patient, considering their characteristics and taking advantage of each therapy line requires more insight in real practice [8, 12, 13]. Real-world studies are needed to generalize results to real-life populations [14, 15].

The objective of the non-interventional REMIX study to evaluate IXA use in real life has been underway in France since IXA became available in 2017 to generate supplementary data derived from use in an unselected population of RRMM patients. The REMIX study is one of the largest prospective, studies to provide real-world evidence (RWE) of the IXA-Rd combination. The study has been designed to assess effectiveness and safety in the treated population as well as to refine the appropriate patient profile to receive the combination.

## Material and methods

### Study design

REMIX was a non-interventional, prospective, multicenter study conducted in France in patients with RRMM who received an oral formulation of IXA combined with lenalidomide and dexamethasone (IXA-Rd) in real-life conditions. The decision to treat with IXA-Rd was at the physician’s discretion. Patient management was performed according to standard of care at each site.

To be eligible, adult patients had to have received IXA-Rd after at least 1 prior line of chemotherapy according to the summary of product characteristics (SmPC) of each product and IXA had to be initiated concomitantly to Rd. If lenalidomide was started more than 6 weeks before IXA, it was considered non-concomitant and the patient excluded from the primary analyses. In France, IXA-Rd was available under compassionate access from August 2017 until October 2018 when IXA became commercially available. Patients were prospectively enrolled during the first four months after starting IXA-Rd. Patients were followed up for at least 24 months (max 49.5 months) or until the end of the study or death, whichever occurred first, according to the site's standard practices.

To ensure a representative patient population, sites participating in the nationwide compassionate access (*n* = 158) were invited to participate in the study. Among these sites, 64 accepted, which included both public and private hospitals located throughout France. Of these, 60 sites actively enrolled patients in the study.

### Study endpoints

The primary endpoint measure was median progression-free survival (mPFS) and PFS rates assessed at 12, 18, 24, and 36 months. PFS was defined as the time interval from the date of first dose of IXA to the date of disease progression or death, whichever occurred first. Secondary endpoints included overall survival (OS) defined as the time interval from the date of first dose of IXA to the date of death, at 12, 18, 24, 36, 42 and 48 months, duration of response (DoR) defined as the time interval between the best response to treatment to progression or death, whichever occurred first among patients with at least a partial response (PR), and endpoints based on the response rates (RR): complete response (CR), very good partial response (VGPR), PR and the stable disease (SD) rates. Overall response rate (ORR) combined CR, VGPR and PR. Safety endpoints included incidences of adverse events (AEs), serious AE (SAEs), treatment-related AEs and SAE, and AE leading to treatment discontinuation.

### Assessment and data collection

Data was collected every 3 months during the first two years, then every 6 months thereafter until study end, as per standard practice. An investigator assessed the therapeutic response including the refractory status to lenalidomide (Twenty-six (6.9%) patients were reported to be lenalidomide-refractory, although this was a non-inclusion criterion) and disease progression according to International Myeloma Working Group (IMWG) criteria [16] as in usual practice at each site (no central review). Safety data were collected for up to 30 days after the last treatment dose administration. Treatment discontinuation was based on the investigator judgment. Post-IXA-Rd data were collected for subsequent therapies description and survival status. Patient cytogenetic abnormalities, Eastern Cooperative Oncology Group performance status (ECOG-PS) and International Staging System (ISS) were not routinely conducted in all sites but were collected at IXA-Rd initiation when available. Comorbidities were estimated with the Charlson's Comorbidity Index and frailty was evaluated using the simplified frailty score based on age, Charlson’s Comorbidity Index and ECOG-PS [17].

All patients provided their consent to participate to the study as per local regulations. Data were remotely monitored regularly during the study and each site was visited at least once by a monitoring Clinical Research Assistant. Monthly safety reconciliation with the sponsor safety database were performed and annual data reviews were organized.

This study was conducted in accordance with the ethical principles of the Declaration of Helsinki [18], the principles of the International Society for Pharmacoepidemiology guidelines for Good Pharmacoepidemiology Practice [19], and in compliance with the General Data Protection Regulation (GDPR) [20]. The protocol was approved by a French Ethical Committee on November 9th, 2017 (N° AU 1381). All patients provided their consent to participate to the study as per local regulations.

### Sample size and statistical analysis

Analyses were performed in the eligible population.

Baseline patient characteristics, response, and safety data were summarized using descriptive statistics. Qualitative data are presented as numbers with the corresponding percentages. While quantitative data are presented as means with standard deviation (SD) and/or median with interquartile range (IQR). The numbers of patients with missing data are indicated. Missing data were not replaced.

Time-to-event analyses (PFS, OS, treatment duration, and DoR) were estimated using the Kaplan–Meier method, the 95% CI were estimated using the Greenwood formulae. Survival differences were compared in subgroups using the log rank test. Patients, still alive, with no disease progression at end of the study were censored at the date of their last disease assessment. Patients without response or progression assessments at the date of database lock were excluded from the analysis based on these data. 

PFS, OS, and ORR were assessed overall and within subgroups: according to the lines of treatment (L2, L3, and L4 +), age groups (< 80 years old versus ≥ 80-years old), frailty (frail versus non frail), prior exposure to lenalidomide, time interval between last lenalidomide and IXA-Rd (≤ 12 months versus > 12 months), renal failure based on creatinine clearance at initiation (> 50 ml/min, 30–50 ml/min, ≤ 30 ml/min), autologous stem cell transplantation (ASCT), comorbidities (Charlson score), and cytogenetic abnormalities at baseline (standard risk (SR) versus high (HR) defined as del(17p) and/or t(4;14) and/or t(14;16)). 

A total of 500 patients were expected to participate in the study. A sample size of 250 patients per subgroup would provide an accuracy of 6.2% in describing the study results.

Statistical analyses were conducted using SAS version 9.4 (SAS Institute Inc., Cary, NC, USA).

## Results

### Patients

The REMIX study enrolled 376 patients who initiated IXA concurrently with Rd between August 2017 and October 2019 in 60 active participating sites: 197 during the compassionate access period and 179 thereafter. 32 patients were excluded from the analysis including 29 patients because lenalidomide was initiated more than 1 month before IXA, 2 patients because IXA-Rd was not initiated and 1 patient who did not complete its inclusion visit. 

Patient demographics and disease characteristics are summarized in Table 1. At IXA-Rd treatment initiation, the median age was 71 years and 69 (18.4%) patients were 80 years or older. Among the 209 patients with available data, 18.2% patients had an ECOG performance status ≥ 2 including 4 patients (1.9%) with an ECOG = 3 (no patient with an ECOG = 4). Also, in the study population, 48.8% were frail and 62.8% had at least one comorbidity. Charlson score, lines of treatment, cytogenetic abnormalities, and the time intervals from diagnosis to IXA-Rd initiation were similar in the age groups except the frailty score (≥ 80 years: 96.7% and < 80 years: 35.9%).

**Table 1:** Patient demographics and disease characteristics

	All patients (*N* = 376)
Median age at IXA-Rd start (in years) (IQR)	71 (65.0–77.5)
≥ 75, *n* (%)	133 (35.4)
≥ 80, *n* (%)	69 (18.4)
Male Sex, *n* (%)	185 (49.2)
Charlson index total score, *n* (%)	
0	246 (65.4)
1–2	100 (26.6)
3–4	21 (5.6)
≥ 5	9 (2.4)
ECOG at IXA-Rd start, *n* (%)	*n* = 209
0	69 (33.0)
1	102 (48.8)
≥ 2	38 (18.2)
Simplified frailty scale at IXA-Rd start, *n* (%)	*n* = 283
Frail	138 (48.8)
Non frail	145 (51.2)
M Protein type,(*n* (%)	
IgG	211 (56.1)
IgA	81 (21.5)
None	58 (15.4)
Other	9 (2.4)
Data not available	20 (5.3)
Light chain type, *n* (%)	
Kappa	257 (68.4)
Lambda	112 (29.8)
Data not available	7 (1.9)
Cytogenetic features at IXA-Rd start, *n* (%)	
Standard-risk cytogenetic abnormalities	167 (44.4)
High-risk cytogenetic abnormalities	45 (12.0)
Data not available	164 (43.6)
Median time since diagnosis (in years)	4.0
Line of treatment at IXA-Rd start, *n* (%)	
L2	227 (60.4)
L3	68 (18.1)
L4 +	81 (21.5)
Creatinine clearance (ml/min) at IXA-Rd start, *n* (%)	*n* = 304
> 50	238 (78.3)
30–50	43 (14.1)
≤ 30	23 (7.6)

### Prior therapy and pre-exposure to lenalidomide

Prior therapies before IXA-Rd start are described Table 2. Most patients, 227 (60.4%), had received only 1 previous line of therapy. IXA-Rd was prescribed second-line in 60.0% of patients, third-line in 18%, and fourth and further lines in 22%. Most patients, 344 (91.7%), were previously treated with bortezomib and 244 (65.1%) had prior immunomodulatory drug therapy, of whom 39.2% had been exposed to lenalidomide and 42.4% to thalidomide. 52 (14%) patients, had received daratumumab, which was only available on compassionate access in France during the study. About half of the patients, 167 (44.5%), had prior autologous stem cell transplantation (ASCT); 53.3% of patients in third or fourth lines of treatment or more versus 38.8% in second lines. Data about refractive status were available only for lenalidomide (*n* = 26 lena-refractory). Prior exposure to lenalidomide was present in 10.6% of patients in second line, 73.5% in third line, and 91.3% in fourth or further lines. Median prior lenalidomide duration was similar whatever the line (16.0–18.0 months) and median duration between the last lenalidomide dose and start of IXA-Rd therapy was 16 months with 59.5% patients having more than 12 months washout period before re-exposure.

**Table 2:** Prior therapy before IXA-Rd start

	All Patients (*N* = 376)	Second Line (*N* = 227)	Third Line (*N* = 68)	≥ Fourth line (*N* = 81)
Prior proteasome inhibitor therapy, *n* (%)	349 (93.1)	210 (92.5)	60 (88.2)	79 (98.8)
bortezomib	344 (91.7)	207 (91.2)	59 (86.8)	78 (97.5)
carfilzomib	28 (7.5)	3 (1.3)	6 (8.8)	19 (23.8)
Prior immunomodulatory drug therapy, *n* (%)	244 (65.1)	105 (46.3)	61 (89.7)	78 (97.5)
lenalidomide	147 (39.2)	24 (10.6)	50 (73.5)	73 (91.3)
pomalidomide	44 (11.7)	1 (0.4)	1 (1.5)	42 (52.5)
thalidomide	159 (42.4)	84 (37.0)	32 (47.1)	43 (53.8)
Prior exposure to other therapy, *n *(%)				
melphalan	170 (45.3)	100 (44.1)	26 (38.2)	44 (55.0)
cyclophosphamide	76 (20.3)	27 (11.9)	15 (22.1)	34 (42.5)
daratumumab	52 (13.9)	10 (4.4)	2 (2.9)	40 (50.0)
bendamustine	26 (6.9)	2 (0.9)	1 (1.5)	23 (28.8)
vincristine	24 (6.4)	3 (1.3)	5 (7.4)	16 (20.0)
doxorubicin	20 (5.3)	1 (0.4)	6 (8.8)	13 (16.3)
panobinostat	3 (0.8)	0 (0.0)	0 (0.0)	3 (3.8)
At least one ASCT during previous therapy, *n* (%)	167 (44.5)	88 (38.8)	37 (54.4)	42 (52.5)
Last line before IXA-Rd exposure, *n* (%)	94 (25.1)	24 (10.6)	46 (67.6)	24 (30.0)
Median duration of exposure to lenalidomide (months)	17.0	18.0	16.0	17.0
Median duration between lenalidomide and IXA-Rd Start (months)	16.0	20.0	9.0	19.0
> 12 months, *n* (%)	78 (59.5)	16 (69.6)	20 (47.6)	42 (63.6)
> 24 months, *n* (%)	42 (32.1)	8 (34.8)	10 (23.8)	24 (36.4)
Refractory to lenalidomide, *n* (%)	26 (6.9)	2 (0.9)	4 (5.9)	20 (25.0)

### IXA-Rd initiation

Most patients (90.4%, *n* = 340) initiated IXA at the full dosage of 4 mg/day, while the remaining 36 patients were prescribed 3 mg/day or less. The starting daily dose of lenalidomide varied from 25 mg in 61.3% of patients (*n* = 228) to 20 mg in 4.0% (*n* = 15), 15 mg in 16.9% (*n* = 63) and 10 mg or less in 17.7% (*n* = 66) of them. Dexamethasone was associated with IXA-R at a daily dose of 40 mg or 20 mg in 52.7% (*n* = 195) and 43.0% of patients (*n* = 159), respectively. 

### Effectiveness

After a median follow-up of 28.7 (min: 0.4 – 49.5) months from patients’ enrolment to end of the study or death, whichever occurs first, 226 of 358 (63.1%) patients had progressed or died. At analysis, 1 patient was lost to follow-up, 17 patients had not been assessed for disease progression but were still alive and were not included in the PFS (*n* = 358) analysis.

**Fig. 1:**
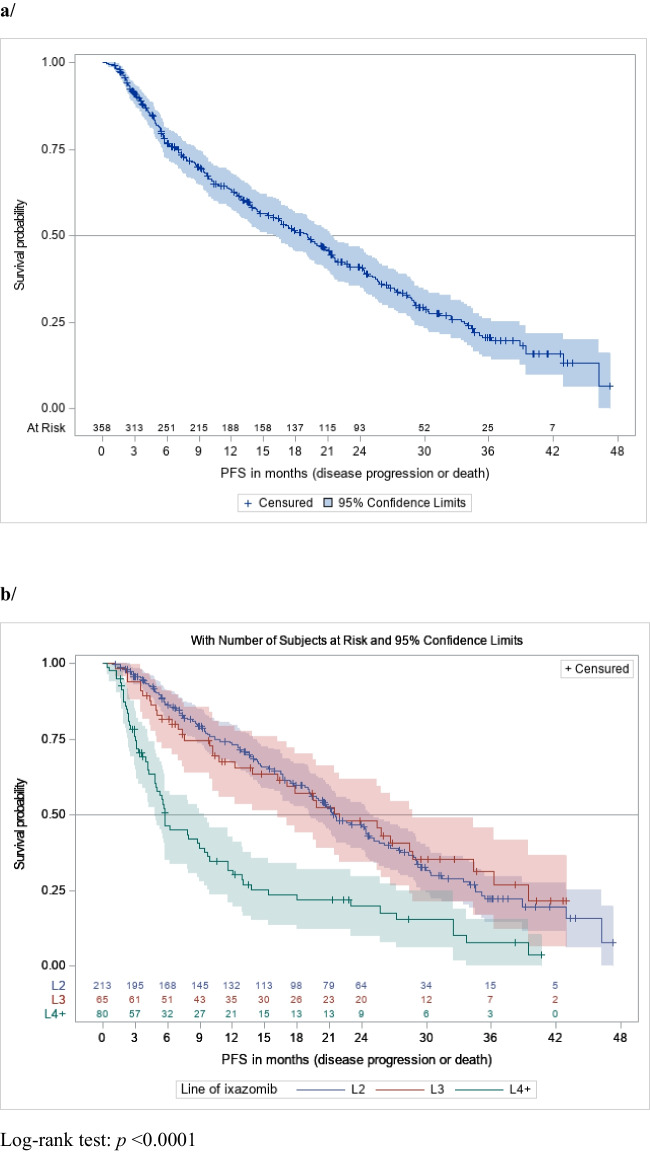

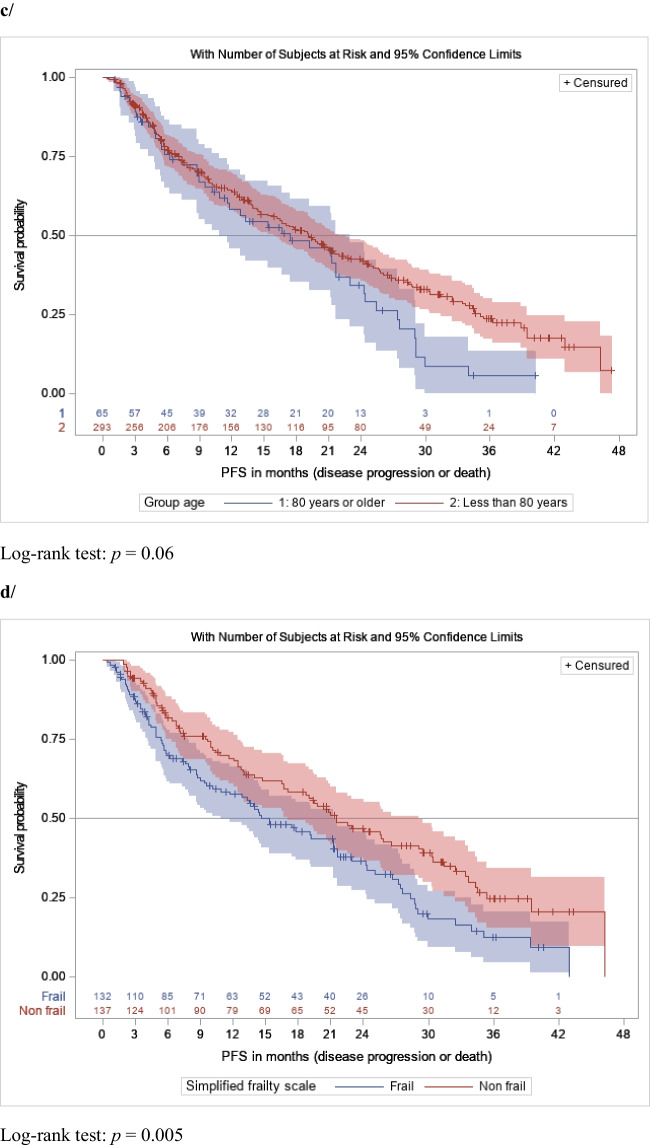
PFS distributions with 95% confidence intervals in **a**/ the overall population; **b**/ patients receiving IXA-Rd as second-line therapy or as third line therapy and beyond; **c**/ patients less than 80 years-old and of 80 years-old and more; **d**/ frail and non-frail patients

The Kaplan–Meier estimates of PFS are shown in Fig. 1. mPFS was 19.1 months (95% CI [15.9–21.5]) in the overall population (Fig. 1a). mPFS was 21.5 months (95% CI [19.2–24.8]) in patients receiving IXA-Rd as second-line treatment, 21.9 months (95% CI [16.2–28.7]) as third-line treatment, and 5.8 months (95% CI [4.8–9.4]) as fourth or further lines of treatment, respectively *p* < 0.01 (Fig. 1b). mPFS was 19.1 months (95% CI [15.9–21.9]) in patients younger than 80 years old and 17.4 months (95% CI [10.8–23.0]) in those 80 years old or older *p* = 0.06 (Fig. 1c). mPFS was significantly lower in frail patients versus non-frail (14.6 months (95% CI [10.8–21.3] versus 21.5 months (95%CI [17.0–29.1]), *p* < 0.01, Fig. 1d). mPFS was similar in patients with and without previous ASCT (19.8 months (95% CI [14.3, 24.8]) and 17.8 months (95% CI [14.4–21.5]) *p* = 0.30) or in comorbidities subgroups (with previous comorbidities: 19.5 months (95% CI [12.8–24.0]) and without comorbidities: 18.8 months (95% CI [15.3–21.9]; *p* = 0.67). Regarding cytogenetic abnormalities, mPFS was 21.2 months (95% CI [14.7–25.6]) in the standard risk group, 19.8 months (95% CI [16.4–29.0]) in the high-risk group and 15.4 months (95% CI [11.6–21.0]) in the group without the evaluation, *p* = 0.07).

Best response rates are detailed in Table 3. The investigator-assessed ORR was 73.1% with IXA-Rd. The best response was CR in 14.5% of patients, VGPR in 30.5%, PR in 28.1% and SD in 10.6% of patients with available response assessment (*n* = 331). ORR was similar in the age groups: 72.4% in those < 80 years old and 76.8% in those ≥ 80 years old. ORR was increased when IXA-Rd was taken second or third line (80.3% and 70%, respectively) and decreased when taken in fourth line or further (54.4%). In the study population, median DoR was estimated at 10.9 months (95% CI [8.7–14.8]).

**Table 3:** Best response and overall survival

	All Patients (*N* = 376)	Second Line (*N* = 227)	Third Line (*N* = 68)	≥ Fourth line (*N* = 81)	< 80 YO (*N* = 307)	≥ 80 YO (*N* = 69)
Best response, *n* (%)	*N *= 331	*N* = 203	*N* = 60	*N* = 68	*N* = 275	*N* = 56
Complete Response (CR)	48 (14.5)	32 (15.8)	10 (16.7)	6 (8.8)	39 (14.2)	9 (16.1)
Very Good Partial Response (VGPR)	101 (30.5)	75 (36.9)	15 (25.0)	11 (16.2)	90 (32.7)	11 (19.6)
Partial Response (PR)	93 (28.1)	56 (27.6)	17 (28.3)	20 (29.4)	70 (25.5)	23 (41.1)
Stable Disease (SD)	35 (10.6)	18 (8.9)	6 (10.0)	11 (16.2)	27 (9.8)	8 (14.3)
Progressive Disease (PD)	54 (16.3)	22 (10.8)	12 (20.0)	20 (29.4)	49 (17.8)	5 (8.9)
Duration of best response (months)	*N* = 242	*N* = 163	*N* = 42	*N* = 37	*N* = 91	*N* = 23
Median (95% CI)	10.9 [8.7; 14.8]	10.9 [8.5; 15.9]	14.7 [6.9; 23.6]	9.1 [3.5; 19.5]	10.8 [7.8; 13.7]	16.1 [5.1; 21.2]
IQR	[3.5; 20.0]	[3.9; 19.6]	[5.0; 23.9]	[2.4; 19.9]	[3.5; 19.6]	[3.3; 22.1]
Overall survival, survival rate % [CI**]**	*N* = 375	*N* = 226	*N* = 68	*N* = 81	*N* = 306	*N* = 69
Median (95% CI)	NE	NE	NE	18.5 [11.0; 33.7]	NE	31.6 [23;.]
12 months	82.2 [78.3; 86.1]	89.2 [85.1; 93.3]	86.6 [78.4; 94.7]	59.2 [48.5; 69.9]	83.8 [79.7; 88.0]	75.0 [64.7; 85.3]
24 months	71.6 [67.0; 76.3]	79.3 [73.9; 84.7]	80.3 [70.7; 89.9]	43.0 [31.9; 54.0]	74.0 [69.0; 79.0]	60.9 [49.1; 72.7]
36 months	58.3 [52.6; 63.9]	63.4 [56.1; 70.8]	68.9 [57.1; 80.7]	35.3 [23.9; 46.7]	61.7 [55.6; 67.8]	41.4 [26.6; 56.2]
42 months	55.4 [49.4; 61.5]	62.3 [54.8; 69.8]	60.9 [46.1; 75.7]	31.8 [19.6; 43.9]	58.5 [51.9; 65]	41.4 [26.6; 56.2]
48 months	52.4 [44.2; 60.5]	57.5 [46.1; 68.9]	60.9 [46.1; 75.7]	. % [.;.]	54.8 [45.5; 64.1]	41.4 [26.6; 56.2]

NE: not estimated

At the time of the present analysis the median OS had not been yet been reached (Online Resource 1). The estimated OS rate was 82.2% (78.3; 86.1) at 12 months, 71.6% (67.0; 76.3) at 24 months, 58.3% (52.6; 63.9) at 36 months, 55.4% (49.4; 61.5) at 42 months and 52.4% (44.2; 60.5) at 48 months. In the subgroup of patients treated in the fourth line or further the median OS was 18.5 months (95% CI [11.0, 33.7]). In patients older than 80 years the median OS was 31.6 months (95% CI [23.0, not reached]).

### Effectiveness and pre-exposure to lenalidomide

**Fig. 2:**
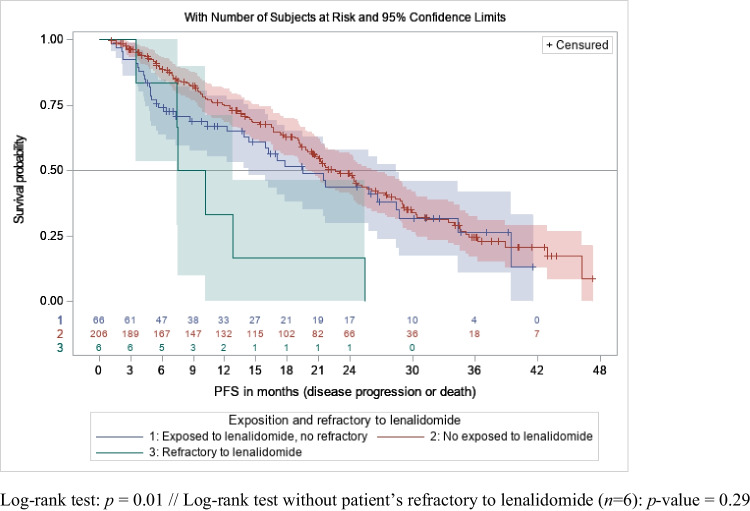
PFS distributions with 95% confidence intervals in patients receiving IXA-Rd in L2 and L3 according to prior exposure to lenalidomide

When focusing on second and third lines (*n* = 272), mPFS was similar in patients previously treated with lenalidomide (mPFS of 19.5 months (95% CI [14.3–28.4]) and in patients not exposed to lenalidomide (mPFS of 22.6 months, 95% CI [20.0–26.7]), *p* = 0.29 (Fig. 2) without any differences in patients’ characteristics in both groups. Those results were similar when the analysis focused on patients of second line (not enough patients to estimate results for third line).

In pre-exposed patients (*n* = 64), when time between last lenalidomide dose and IXA-Rd start was ≤ 12 months, mPFS was 7.4 months (95% CI [4.9–17.8]). It was 25.8 months (95% CI [15.9-not reached]) when this washout period exceeded 12 months (*p* = 0.0043, Online Resource 2).

### Safety

A dose reduction was observed in 99 patients (26.4%) for IXA and in 129 patients (34.4%) for lenalidomide during treatment. Treatment temporary suspensions were reported in 83 patients (22.1%) with IXA and in 80 patients (21.3%) with lenalidomide. At analysis, the median duration of treatment with IXA was 12.4 months. At final analysis, 278 (74.1%) had permanently discontinued IXA and 215 (57.3%) lenalidomide. IXA discontinuation was due to toxicity in 21% (79/376) of patients and progression in 34.6% (130/376) of patients. Respectively, 69.6% and 75.2% of patients ≥ 80 years and < 80 years discontinued IXA of whom 21.7% (15/69) and 20.9% (64/306) due to adverse events. 

AEs were reported in 294 patients (78.2%) treated with IXA-Rd including 54.3% of patients with SAE and 40.7% with treatment-related AE. The most frequently treatment-related AEs (> 10 patients) were diarrhea (13.9%), thrombocytopenia (12.6%), nausea (8.5%), asthenia (7.1%), anemia (4.4%), neutropenia (4.4%), vomiting (4.1%), peripheral neuropathy (4.1%) and unspecified cytopenia (3.4%). The incidence of AE, AE related to treatment, SAE, and SAE related to treatment are 77.2%, 41.7%, 54.1% and 16.0% in patients < 80 and 82.6%, 36.2%, 55.1%, 11.6% in patients ≥ 80-year-old. Overall, most frequent SAE included thrombocytopenia (12.2% of patients with at least one AE), plasma cell myeloma (9.5%), death (7.8%), neutropenia (5.8%), general physical health deterioration (5.4%), diarrhea (4.4%) and anemia (4.4%) as presented in Table 4.

**Table 4:** Adverse events reported during the study in patients treated with IXA-Rd

	Patients (*N* = 376)	
	Patients with at least one AE (*N* = 294)	Patients with at least one treatment-related AE (*N* = 153)
Patients with:		
any AE, *n* (%)	294 (78.2)	153 (40.7)
any serious AE, *n* (%)	204 (54.3)	57 (15.2)
any AE leading to death, *n* (%)^*^	67 (17.8)	1 (0.3)
Most frequent AE (*n* patients, %):		
Diarrhea	77 (26.2)	41 (13.9)
Thrombocytopenia	68 (23.1)	37 (12.6)
Asthenia	44 (15.0)	21 (7.1)
Neutropenia	35 (11.9)	13 (4.4)
Nausea	34 (11.6)	25 (8.5)
Anemia	33 (11.2)	13 (4.4)
Plasma cell myeloma	29 (9.9)	1 (0.3)
Death	23 (7.8)	0 (0.0)
Neuropathy peripheral	21 (7.1)	12 (4.1)
Vomiting	19 (6.5)	12 (4.1)
General physical health deterioration	17 (5.8)	1 (0.3)
Cytopenia	16 (5.4)	10 (3.4)
Constipation	15 (5.1)	9 (3.1)
Fatigue	14 (4.8)	8 (2.7)
Oedema peripheral	14 (4.8)	1 (0.3)
Pancytopenia	14 (4.8)	8 (2.7)
Back pain	14 (4.8)	2 (0.7)
Muscle spasms	13 (4.4)	3 (1.0)
Paresthesia	11 (3.7)	0 (0.0)
Acute kidney injury	11 (3.7)	1 (0.3)
Bronchitis	10 (3.4)	4 (1.4)
Insomnia	10 (3.4)	2 (0.7)

^*^There were 21 patients with AE leading to death while being treated with IXA-Rd

### Subsequent therapy

After IXA-RD treatment discontinuation, in the 177 patients with available data, subsequent therapies mostly comprised pomalidomide (*n* = 99, 55.9%), daratumumab (*n* = 91, 51.4%), carfilzomib (*n* = 63, 35.6%), bortezomib (*n* = 60, 33.9%) and cyclophosphamide (*n* = 53, 29.9%).

## Discussion

The REMIX study is the largest prospective, real-world study to evaluate orally administered combination IXA-Rd in patients with RRMM and confirms the efficacy and safety of the IXA-Rd triplet oral regimen with a mPFS of 19.1 months in RRMM patients with in median 1 prior line of treatment and a proportion of 39% prior exposure to lenalidomide. 

The REMIX study confirms that IXA-Rd is safe and effective in elderly patients. The study included a high proportion of patients older than 75 years (35%) and 80 years (18%), and the median age (71 years) was higher than in any other real-world study [21–25], as presented in Table 5. The efficacy in terms of mPFS and ORR remains meaningful in these elderly patients, particularly in those aged 80 years and over (mPFS of 17.4 months, ORR of 76.8%); the mPFS was similar in younger patients (*p* = 0.06). The median OS was reached in the elderly subgroup, however, as expected, younger patients had not yet reached the median overall survival. This is important because a well-tolerated, oral triplet regimen is particularly advantageous for older patients compared to other available treatment options, which are more intensive and require hospital administration. Patients older than 80 years are generally excluded from clinical trials and there are few data regarding their outcomes in the literature. However, these patients are crucial, as they represent a quarter of the patients seen in routine clinical practice. To our knowledge, this is the first study to publish this insight into older RRMM patients and the demonstrated benefits of the IXA-Rd triplet mean this could be an alternative treatment option for a population that clinicians deal with daily [6, 8].

**Table 5:** The REMIX study and other IXA-Rd papers

Reference	Name of the study	Design	Country	Number of patients	Patient characteristics	Effectiveness	Safety	Subgroups of interest
Moreau P et al*.* [4] / Richardson et al. [26]	TOURMALINE-MM1	Clinical trial	26 countries	722 patients (360 in IXA-Rd group)	Median age: 66 years, % > 65: 53% L2 (62%), L3 (27%), L4 (11%) Previously exposed to R: 12%	ORR: 78% ≥ VGPR: 48% mPFS: 20.6 months mPFS > 75 years: 18.5 mPFS ISS III: 18.4 months mPFS high-risk cytogenetics: 21.4 months mOS: 53.6 months	Discontinuation due to toxicity: 17%	Age, ISS stage, cytogenetic risk, number of prior therapies, prior exposition to IP and IMID, refractory to last therapy, relapsed or refractory
Macro M et al. 2023 (present study)	REMIX study	Real-world prospective study	France	376 patients across 60 sites	Median age: 71 years, %80 + : 18% L2 (60%), L3 (18%), L4 + (22%) Previously exposed to R: 39.2%	ORR: 73% ≥ VGPR: 45% mPFS: 19.1 months L2 and L3: 22 months vs L4 + : 6 months ≥ 80y: 17 months vs 19 months in < 80y frail: 15 months vs 22 months in non-frail mOS not reached	Discontinuation due to toxicity: 21%	Age, frailty, line of treatment, renal failure, comorbidities, previous ASCT, prior exposure to R
Varga G et al. [21]	-	Real-world retrospective study	Hungary	77 patients treated at 7 centers	Median age: 66 years L2 (27%), L3 (35%), L4 (39%)	ORR: 67% ≥ VGPR: 23% mPFS: 11.4 months mPFS not reached in L2, was 10 months in L3 and 8.8 months in L4 No difference according ISS and cytogenetic profile	No permanent drug interruptions due to AEs	ISS classification Cytogenetic profile
Cohen YC et al. [22]	-	Real-world retrospective study	Israel	78 patients across 7 sites	Median age: 68 years L2 (64%), L3 (19%), L4 + (17%) Previously exposed to R: 26%	ORR: 88% ≥ VGPR: 45% mPFS: 24 months mPFS not reached vs 20.2 months for age ≤ 65 vs > 65, respectively mOS not reached	Discontinuation due to toxicity: 14%	Age (≤ 65) No effect on PFS was found for gender, BSA, ixazomib line number, diagnosis paraprotein and involved light chain, cytogenetic risk, ISS, presence of CRAB or EMD levels above ULN, prior drug exposure (IMiDs, PIs), and prior ASCT
Terpos E et al. [23]	-	Real-world retrospective study	Greece, the UK, and the Czech Republic	155 patients who received IXA via early access programs	Median age: 68 years L2 (51%), L3 (28%), L4 + (21%) Previously exposed to R: 17%	ORR: 74% (76.5% in L2, 71.2% in L3 +) ≥ VGPR: 35% mPFS: 27.6 months L2: 27.6 months L3 + : 19.9 months prior exposure to R: mPFS 4.8 months (n = 26) no prior exposure to R: 27.6 (n = 129)	Discontinuation due to adverse events/toxicity: 9%	Gender Prior ASCT Length of ixazomib exposure Prior IMID exposure
Minarik J et al*.* [24]	-	Real-world prospective study	The Czech Republic	127 patients	Median age: 66 years L2 (58%), L3 (24%), L4 + (19%) Previously exposed to R: 17% (6% refractory)	ORR: 73% ≥ VGPR: 33.3% mPFS: 17.5 months L2: 32.8 months L3: 23.1 months L4: 9.7 months L5 + : 5 months > 75 years: 11.1 months mOS: 36.6 months	Discontinuation due to toxicity: 3.1%	Age, ISS stage, ASCT, cytogenetics, maximal treatment response, and pretreatment
Hajek R et al. [25]	-	Real-world study	13 countries (INSIGHT MM and the Czech RMG)	263 patients	Median age: 68 years with 15% > 75 years L2 (44%), L3 (35%), L4 + (21%) Previously exposed to R: 27% (7% refractory)	ORR: 73% ≥ VGPR: 37% mPFS: 21 months L2: 26 months L3: 24 months L4: 14 months	Discontinuation due to AEs: 32%	Line of treatment

Frail patients included in the REMIX study also benefited from IXA-Rd treatment. Although mPFS was shorter in this subgroup than non-frail patients (14.6 months versus 21.5 months, *p* < 0.01), this result is nevertheless positive considering the acceptable tolerance profile, which is difficult to compare as frail patients are often excluded from trials. This means IXA-Rd could also be an interesting alternative for frail patients. 

Overall, the efficacy of IXA-Rd in a real-world situation (mPFS of 19.1 months, ORR of 73%) was similar to the controlled, registration study TOURMALINE-MM1 (mPFS of 20.6 months, ORR of 78%) [4]. Yet, the REMIX population was older (median age, 71 years) compared with TOURMALINE-MM1 (median age 66 years), and included more patients with advanced disease (L4 +) 21.5% compared with 11% in TOURMALINE-MM1 and an ECOG > 1 (18.2%) compared with 5% in TOURMALINE-MM1. Importantly, more patients in the REMIX study had been previously exposed to lenalidomide (39.2%) than in TOURMALINE-MM1 (12%) or bortezomib (REMIX 91.7%; TOURMALINE MM1 69%). Also, some REMIX patients had previously received carfilzomib (7.5%), pomalidomide (11.7%) or daratumumab (13.9%) unlike TOURMALINE MM1. Thus, patients were more extensively poly-treated than in TOURMALINE-MM1, which is recognized to have a negative impact on effectiveness. Moreover, 49.8% of the REMIX patients were frail and two third had comorbidities in contrast to the TOURMALINE-MM1 in which frail patients were excluded. The majority of real-world studies conducted are retrospective with small study sample. The mPFS varied from 11.4 to 27.6 months. Patients are younger than in REMIX study and less previously exposed to lenalidomide (maximum: 27%). 

The mPFS of patients in the REMIX study treated with IXA-Rd in L2 (21.5 months) and in L3 (21.9 months) were longer than that of patients in L4 and above (5.8 months), *p* < 0.01. This is in line with TOURMALINE-MM1 and most real-world studies [21–25]. Similarly, the best treatment response (CR and VGPR) occurred more frequently in L2 and L3 than in subsequent lines. As with TOURMALINE-MM1, efficacy remains high in second relapse (L3) and is equivalent to first relapse (L2). In practice, these results are not surprising, and it is now recognized that treatment benefits are reduced at advanced stages of the disease [23, 25, 27]. 

In the REMIX study, a large proportion of patients were pre-exposed to lenalidomide before initiating IXA-Rd. This was especially true for L3 (73.5%) and L4 + (91.3%) patients but rarer for L2 patients (10.6%). It is noteworthy that in this study, pre-exposure to lenalidomide in non-refractory second- and third-line patients is not associated with reduced IXA-Rd efficacy, with mPFS remaining equivalent in both groups (19.5 months versus 22.6 months, *p* = 0.29). For this population, re-using lenalidomide may be of benefit to those whom are sensitive to lenalidomide. Few data are available on lenalidomide pre-exposure, as few patients in TOURMALINE-MM1 had been pre-exposed to lenalidomide, preventing this association from being studied [4, 28]. Results from other real-world studies suggest the benefit may be limited in lenalidomide-pre-treated patients [23]. However, as lenalidomide exposure increases with advanced disease, it is difficult to discern between the real impact of lenalidomide pre-exposure and late disease relapse. Further analysis of REMIX data suggests that a washout period of approximately 12 months between prior lenalidomide exposure and IXA-Rd initiation may improve IXA-Rd efficacy. However, these results should be interpreted with caution, as the washout period duration may be related to other factors such as response to the previous line which are not considered in this analysis.

In addition to lenalidomide pre-exposure, L4 patients had also been exposed to multiple immunomodulatory drugs (IMiD), proteasome inhibitors (PI) and anti-CD38s. Specifically, half of these patients had received pomalidomide or daratumumab and nearly a quarter had received carfilzomib. Conversely, very few L2 or L3 patients had received these immunomodulatory treatments. The multiple exposure of L4 + patients to various agents may reflect the RRMM resistance to treatment and is expected to be associated with the lower IXA-Rd efficacy in this subgroup. 

Dose reductions or treatment interruptions for IXA-Rd are reported for approximately one quarter of patients, similarly with IXA or lenalidomide. Treatment discontinuation related to AEs was noted in 24.5% of patients, in this frailer and older population, which is slightly higher than TOURMALINE-MM1 (17%). As in other studies, the most frequently reported AEs were digestive or hematological, with no new signals identified [23–25, 29]. Unlike most other real-world studies, the REMIX study is prospective and is therefore likely to be more comprehensive and accurate in reporting AEs during follow-up than in retrospective studies.

The limitations of the REMIX study are those inherent to real-world observational studies, notably relating to treatment response or progression assessments, which are assessed by the investigator. Frailty score calculation was based on ECOG-PS if patients were ≤ 80 years, which is less collected in routine clinical practice than in clinical trials. Due to missing data on ECOG-PS, the simplified frailty score was only available for 283 patients (75.0%) The prospective patient recruitment at the start of treatment does not predict the response to treatment and limits the impact of this bias on the efficacy assessment. The representativeness of the patients recruited is still questionable even though the centers were encouraged to propose the study to all their eligible patients. Lastly, the study started when IXA became available in a compassionate program in France. A total of 500 patients were expected to participate in the study with a sample size of 250 patients per subgroup. Even if the total of 500 patients was not reached, an accuracy of 5% was sufficient to estimate the survival analyses and the proportion of patients was similar in each subgroup (*N* = 197 during the compassionate access period before the treatment was marketed and reimbursed and *N* = 179 thereafter), is in line with what was intended. Sensitivity analyses conducted to identify potential selection bias related to the compassionate program showed that those patients recruited during the compassionate access period were similar to those recruited afterwards, although those patients recruited during compassionate access were slightly younger. This can be explained contextually and historically by the treatments available to clinicians during the compassionate program.

In conclusion, the results from the REMIX study are consistent with the TOURMALINE-MM1 results and confirms the benefit of the all-oral IXA-Rd triplet in real life, particularly in early relapse. It reveals that pre-exposure to lenalidomide in non-refractory second and third-line patients is not associated with reduced efficacy and suggests beneficial re-use in early relapses. While many treatments are available to the clinician to manage RRMM patients, REMIX demonstrates the value of the oral IXA-Rd in an elderly population (> 80 years) in which efficacy and acceptable tolerance (they do not experience higher rates of AE or treatment discontinuation) are maintained.

## Supplementary information

Below is the link to the electronic supplementary material.

Supplementary file1 (DOCX 33 KB)

Supplementary file2 (DOCX 39 KB)

## Data Availability

The datasets generated during and/or analysed during the current study are available from the corresponding author on reasonable request.

## Abbreviations

*AEs*: Adverse Events
*ASCT*: Autologous Stem Cell Transplantation
*CR*: Complete Response
*DoR*: Duration of Response
*ECOG*: Eastern Cooperative Oncology Group performance status
*HR*: High Risk
*ISS*: International Staging System
*IMIDs*: Immunomodulatory drugs
*IQR*: Interquartile range
*IXA*: Ixazomib
*IXA-Rd*: Ixazomib-Rd
*Rd*: Lenalidomide and dexamethasone
*mPFS*: Median Progression Free Survival
*ORR*: Overall Response Rate
*OS*: Overall Survival
*PR*: Partial Response
*PI*: Proteasome inhibitors
*RWE*: Real-world evidence
*RRMM*: Relapsed/Refractory Multiple Myeloma
*RR*: Response Rate
*SAEs*: Serious AE
*SD*: Stable Disease
*SR*: Standard Risk
*VGPR*: Very Good Partial Response
