# Supplementary material for: Real-world effectiveness of ixazomib combined with lenalidomide and dexamethasone in relapsed/refractory multiple myeloma: the REMIX study
Source: Ann Hematol. 2023 Jun 10;102(8):2137–51. doi: 10.1007/s00277-023-05278-3 (PMC10344838; doi:10.1007/s00277-023-05278-3)

**Title:** Real-world effectiveness of ixazomib combined with lenalidomide and dexamethasone in relapsed/refractory multiple myeloma: the REMIX study

**Journal name:** Annals of hematology

**Author names**

M. MACRO (MM)1*, C. HULIN (CH)2*, L. VINCENT (LV)3, A. CHARVET-RUMPLER (ACR)4, L. BENBOUBKER (LB)5, C. CALMETTES (CC)6, A-M. STOPPA (AMS)7, K. LARIBI (KL)8, L. CLEMENT-FILLIATRE (LCF)9, H. ZERAZHI (HZ)10, F HONEYMAN (FH)11, V. RICHEZ (VR)12, F. MALOISEL (FM)13, L. KARLIN (LK)14, J. BARRAK (JB)15, C. CHOUAID (CC)16, X. LELEU (XL)17

*** First co-authors**

1 Margaret Macro: IHBN - CHU de Caen, Caen, France

2 Cyrille Hulin : CHU Bordeaux - Hôpital Haut Leveque, Pessac, France

3 Laure Vincent : CHU de Montpellier - Hôpital Saint-Eloi, Montpellier, France

4 Anne Charvet-Rumpler : CHU de Besançon - Hôpital Jean Minjoz, Besançon, France

5 Lotfi Benboubker : CHRU de Tours - Hôpital Bretonneau, Tours, France

6 Claire Calmettes : CH de Périgueux, Périgueux, France

7 Anne-Marie Stoppa : Institut Paoli Calmettes, Marseille, France

8 Kamel Laribi : CH Le Mans, Le mans, France

9 Lauriane Clément-Filliatre : Clinique Louis Pasteur, Essey-lès-Nancy, France

10 Hacene Zerazhi : CH d’Avignon, Avignon, France

11 Fressia Honeyman : CHU de Saint-Etienne, Saint-Etienne, France

12 Valentine Richez : CHU de Nice - Hôpital de l'archet, Nice, France

13 Frédéric Maloisel : Clinique Sainte-Anne, Strasbourg, France

14 Lionel Karlin : Hospices Civils de Lyon, Pierre Bénite, France

15 Joelle Barrak : Takeda France, Paris, France

16 Christos Chouaid : CHI de Créteil, Créteil, France

17 Xavier Leleu : CHU de Poitiers, Poitiers, France

**Corresponding author:** Margaret Macro, [macro-m@chu-caen.fr](mailto:macro-m@chu-caen.fr)

**Online Resource 1: Overall survival distribution with 95% confidence intervals in patients receiving IXA-Rd**


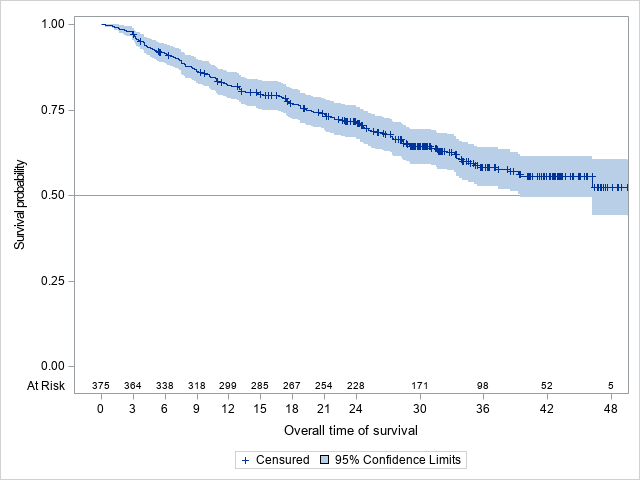

Supplement: Supplementary file 1 — Supplementary file1 (DOCX 33 KB) [file 277_2023_5278_MOESM1_ESM.docx]
